# Supplementary material for: Multi-scale dynamics influence the division potential of stomatal lineage ground cells in Arabidopsis
Source: Nat Commun. 2025 Mar 17;16:2612. doi: 10.1038/s41467-025-57730-9 (PMC11914061; doi:10.1038/s41467-025-57730-9)
Supplement: Supplementary file 1 — Supplementary Information [file 41467_2025_57730_MOESM1_ESM.pdf]

Supplementary data for:

Multi-scale dynamics influence the division potential of stomatal lineage ground cells in *Arabidopsis*

Hannah F. Fung[1,2], Gabriel O. Amador[3], Renee Dale[4], Yan Gong[1,5], Macy Vollbrecht[1], Joel M. Erberich[1], Andrea Mair[2], Dominique C. Bergmann[1,2]*

[1]Department of Biology, Stanford University, Stanford, CA 94305, USA
[2]Howard Hughes Medical Institute, Stanford, CA 94305, USA
[3]Department of Developmental Biology, Stanford University, Stanford, CA 94305, USA
[4]Donald Danforth Plant Science Center, St. Louis, MO 63132, USA
[5]Department of Organismic and Evolutionary Biology, Harvard University, Cambridge, MA 02138, USA
*Corresponding author: dbergmann@stanford.edu

Contents:

Supplementary Figures 1-7

Supplementary Tables 1-5

a

Features tested for predictive value in decision tree analysis

- Time of birth
- X, Y-coordinates
- Birth area of SLGC (μm^2)
- Birth area of sister meristemoid (μm^2)
- Asymmetry of birth division = (SLGC – meristemoid area)/(SLGC + meristemoid area)
- Major axis: length of major axis
- Minor axis: length of minor axis
- Circularity = 4π*area/perimeter^2
- Aspect ratio = major axis/minor axis
- Roundness = 4*area/(π*(major axis)^2)
- Solidity = area/convex area
- Mother identity (meristemoid or SLGC)
- Sister behaviour
  - divided asymmetrically (ACD)
  - differentiated
  - neither divided nor differentiated
- Number of adjacent, non-sister stomatal precursors
  - meristemoids, GMCs, or stomata

b

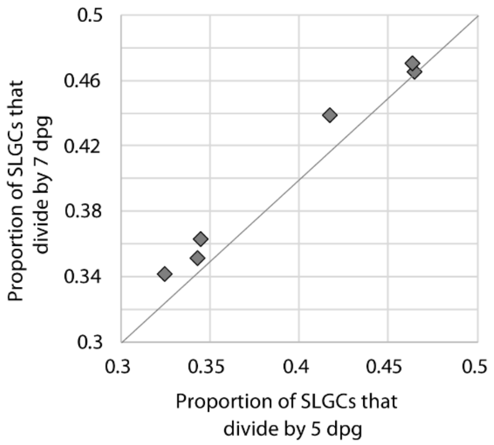

c

Effective α	Nodes	Maximum depth	Accuracy
0	13	4	73.7%
0.03488372	9	3	68.4%
0.06659619	7	3	68.4%
0.07475083	5	2	78.9%
0.07539716	3	1	73.7%
0.09423472	1	0	73.7%

d

Feature	Importance score
Birth size	0.122385
Time of birth	0.121212
Major axis	0.116688
Circularity	0.090541
Minor axis	0.077721
Y-coordinate	0.071104
Solidity	0.066173
Sister meristemoid size	0.063194
Division asymmetry	0.060259
X-coordinate	0.056747
Roundness	0.053900
Aspect ratio	0.050392
Sister behaviour<br>• ACD<br>• differentiation<br>• neither ACD nor differentiation	 • 0.020444<br>• 0.003801<br>• 0.014964
Number of adjacent, non-sister stomatal precursors	0.007750
Mother identity	0.002725

Supplementary Fig. 1 | Additional details on the time-lapse and decision tree analyses. a List of features at birth that were tested for predictive value in the decision tree analysis. **b** Proportion of SLGCs born on 3-dpg that divided by 7-dpg vs. proportion of cells that divided by 5-dpg. The vast majority of cells divided between 3- and 5-dpg. **c** The number of nodes, maximum depth, and accuracy for different cost complexity parameter values (effective α). The parameter value that maximized testing accuracy is highlighted in yellow. **d** The feature importance scores (total reduction in Gini impurity) for all features tested. These values were derived from a random forest classifier with 1,000 trees. Source data are provided in Source Data file 1.

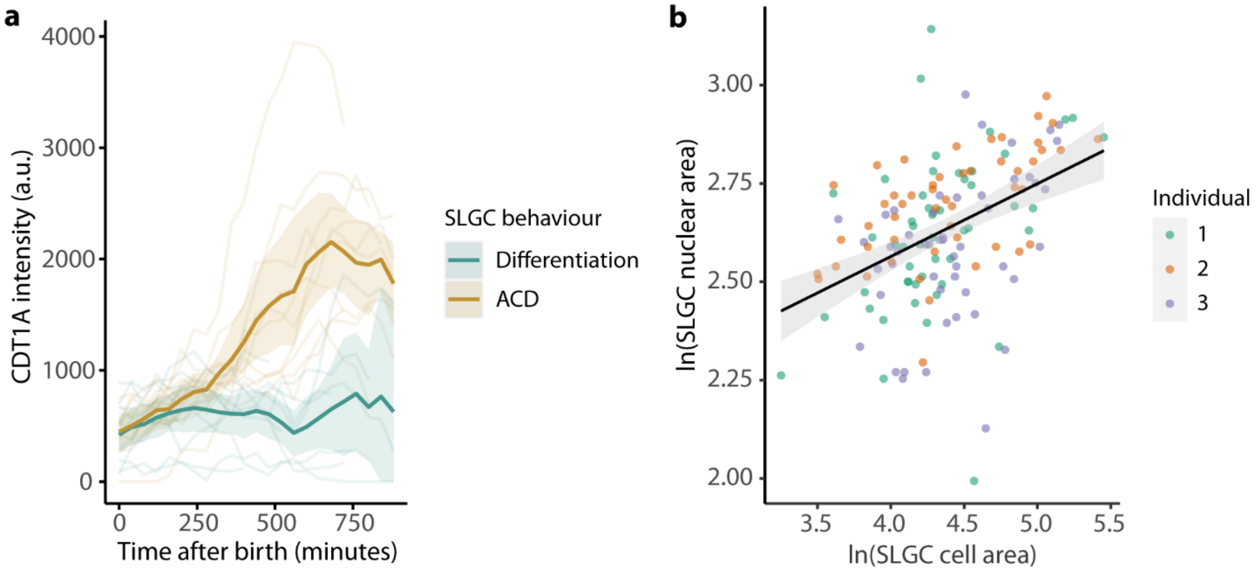

Supplementary Fig. 2 | CDT1A dynamics after birth and the relationship between cell and nuclear areas. a CDT1A-CFP (from the PlaCCI reporter line[46]) intensities over time, coloured by behaviour. In dividing cells (ACD), CDT1A-CFP increased ~300 minutes after birth. Lines and bands are means and bootstrapped 95% confidence intervals. $N = 20$ cells, 10 cells per behaviour. **b** SLGC nuclear area vs. cell area. Axes are ln-transformed. The black line and grey band are linear model predictions and 95% confidence intervals. $N = 3$ individuals, 50 cells per individual. Source data are provided in Source Data file 1.

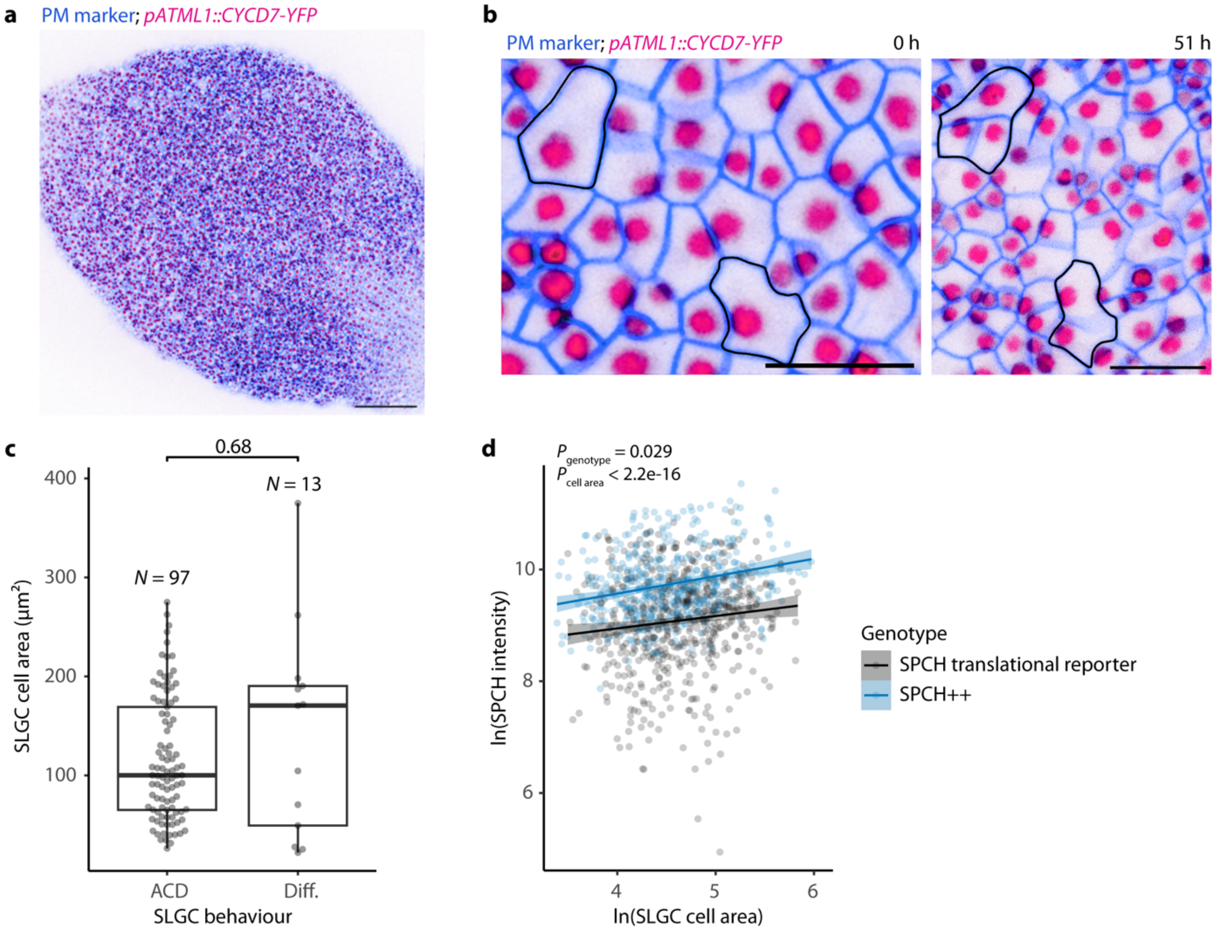

Supplementary Fig. 3 | The epidermis-specific expression of a D-type cyclin induces large SLGCs to divide. a Micrograph of a 3-dpg cotyledon expressing a plasma membrane (PM) marker and the D-type cyclin CYCD7;1 (CYCD7) under the epidermis-specific *ATML1* promoter (*pATML1::CYCD7;1-YFP*). Scale bar: 100 µm. **b** The *pATML1::CYCD7;1-YFP* construct was present in all epidermal cells at 3-dpg (left). The same region 51 hours later (right). Large, dividing SLGCs are outlined in black. Scale bar: 20 µm. **c** Cell areas of SLGCs that divided (ACD) or differentiated (Diff.) in a 3-dpg cotyledon expressing *ATML1p::CYCD7;1-YFP*. The *P*-value is from an unpaired two-sided Wilcoxon rank sum test. $N = 110$ cells. **d** SPCH intensity vs. cell area in *spch-3* cotyledons expressing a SPCH translational reporter or a *pSPCH::SPCH-YFP* transgene that overproduces SPCH (SPCH++). Axes are ln-transformed. *P*-values are from a mixed-effects model with genotype and cell area as fixed effects and individual as a random effect. SPCH translational reporter: $N = 6$ individuals; 85, 85, 93, 94, 102, 154 cells per individual. SPCH++: $N = 4$ individuals; 97, 98, 107, 119 cells per individual. Source data are provided in Source Data file 1.

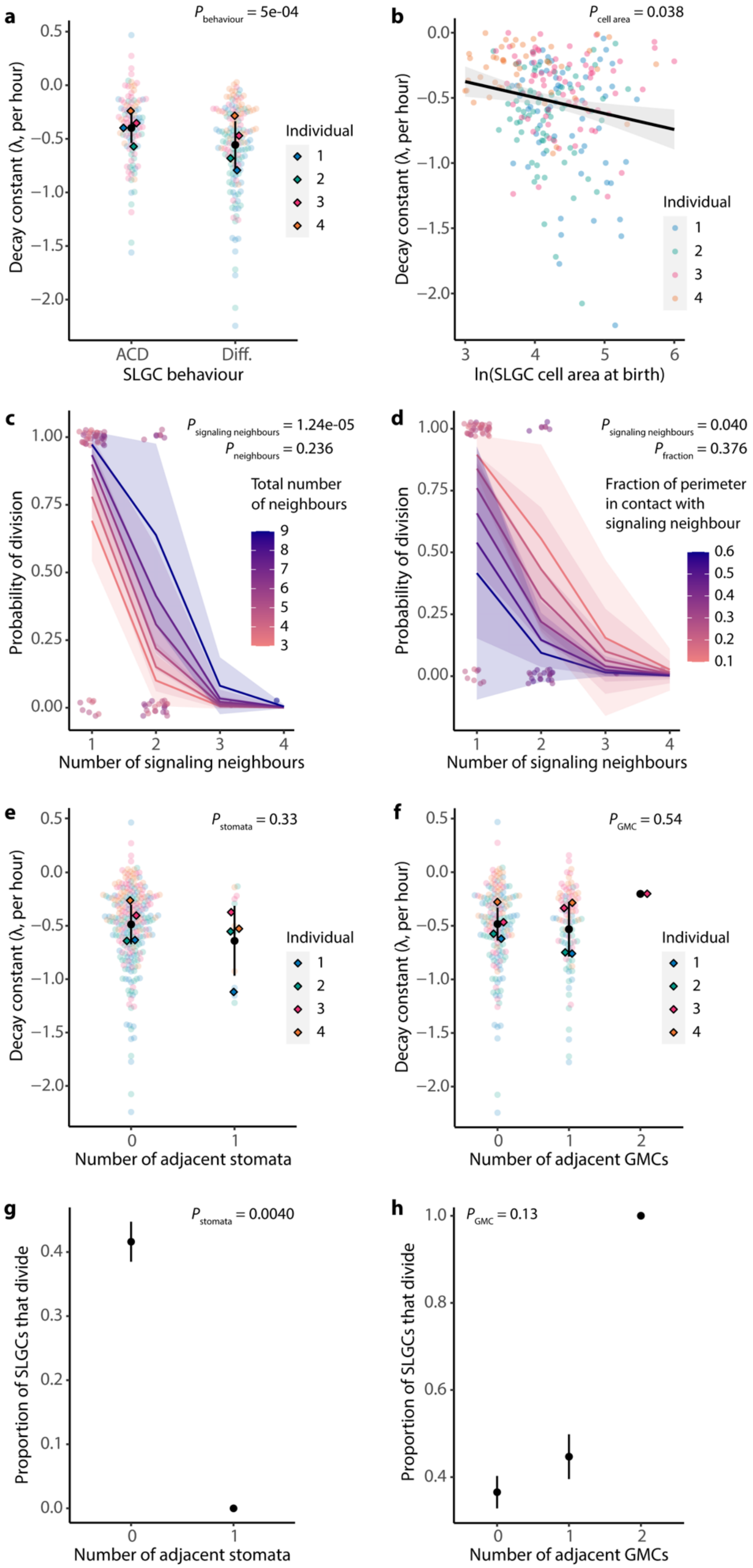

Supplementary Fig. 4 | The effect of signaling neighbours on SPCH degradation rate is not explained by the number of adjacent stomata or GMCs. a The decay constant (how fast SPCH is degraded) in dividing (ACD) or differentiating (Diff.) cells. The more negative the constant, the higher the degradation rate. **b** Decay constant vs. cell area at birth (ln-transformed) of cells where SPCH levels declined. The black line and grey band are linear model predictions and 95% confidence intervals. **c-d** Multiple logistic regressions of the probability of division on the number of signaling neighbours and the total number of neighbours (**c**) or the fraction of the cell perimeter in contact with a signaling neighbour (**d**). The data points only take on *Y*-values of 0 (no division) or 1 (division); they are jittered vertically and horizontally to avoid overplotting. Lines and bands are logistic model predictions and standard errors. *P*-values are from the logistic regressions. *N* = 75 cells. **e-f** The decay constant by the number of adjacent stomata (**e**) or GMCs (**f**). **g-h** The proportion of SLGCs that divided by the number of adjacent stomata (**g**) or GMCs (**h**). **a**,**e-h** Black circles and lines are means and standard deviations. **a**,**b**,**e-h** *P*-values are from mixed-effects models with individual as a random effect (**a-b**,**e-f**) or chi-squared tests for trend in proportions (**g-h**). *N* = 4 individuals; 48, 61, 73, 75 cells per individual. Source data are provided in Source Data file 1.

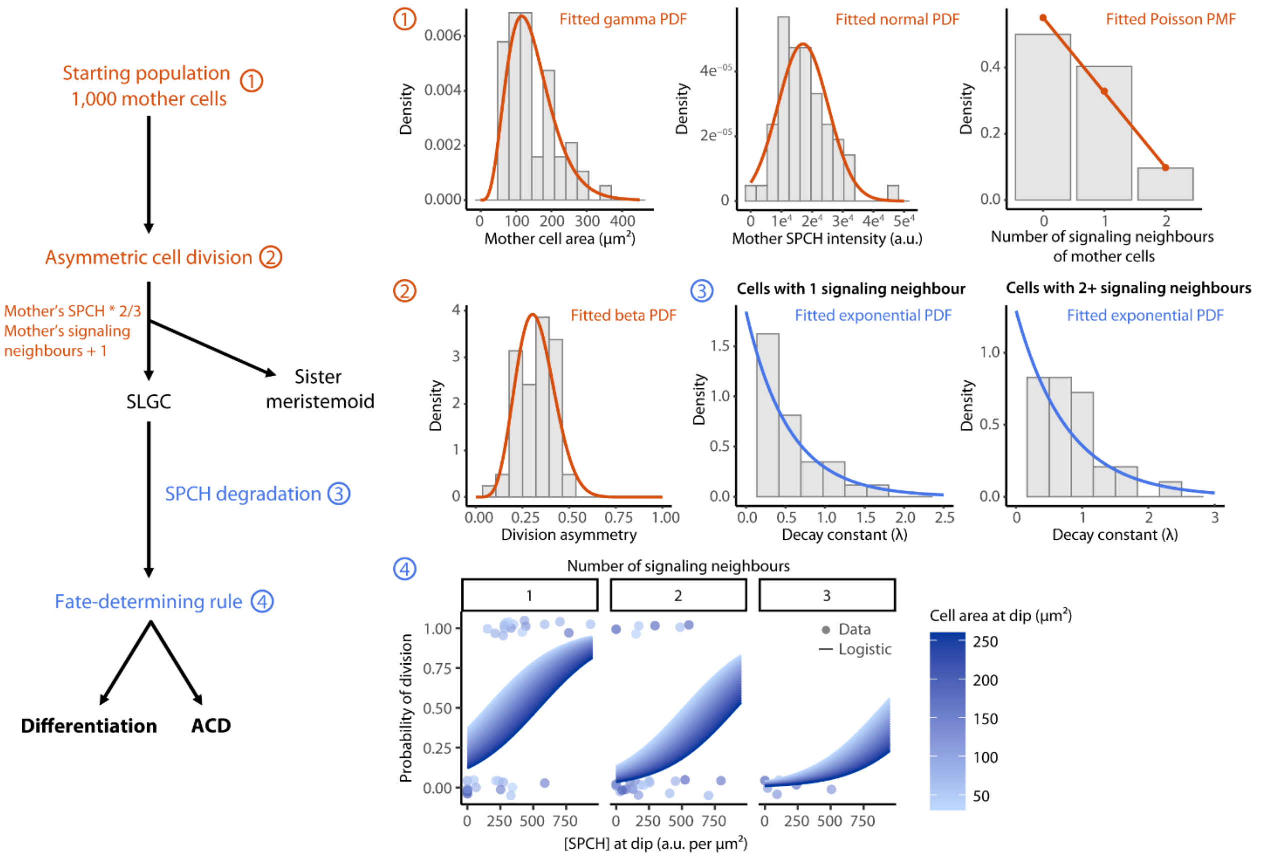

Supplementary Fig. 5 | Diagram of the lineage decision tree model with the best performance overall. The model begins with 1,000 mother cells, each with a randomly drawn starting size, SPCH intensity, and number of signaling neighbours (1; see Supplementary Table 2 for fitted parameters). The mother cells divide with an asymmetry parameter randomly drawn from a beta distribution (2), each forming a smaller meristemoid and a larger SLGC. The asymmetry parameter is calculated as 1 – SLGC cell area/mother cell area. Based on empirical measurements (Supplementary Fig. 6), each SLGC is assumed to inherit two-thirds of its mother's SPCH intensity and to have one signaling neighbour more than its mother (i.e. the newly generated sister meristemoid). After birth, SPCH is degraded at a rate that is proportional to the number of signaling neighbours. Specifically, the decay constant (λ) is randomly drawn from one of two exponential distributions, fitted to the decay constants of cells with 1 and 2+ signaling neighbours, respectively (3). Finally, the cells undergo division or differentiation according to a multiple logistic with cell size, SPCH concentration ([SPCH]), and signaling neighbours as predictors (4). The elements in orange are fixed across models, while the elements in blue are variable. We performed model selection across different modes of SPCH degradation and fate determination to identify the model that best recapitulated the data (see Methods and Supplementary Table 4). All distribution parameters and logistic model parameters were estimated from the data and fed into the decision tree models. The empirical data (grey) and fits (orange or blue) were generated for each of four cotyledon time-lapses and are shown here for individual #1. The source data and code can be found at https://doi.org/10.6084/m9.figshare.27931686.v1.

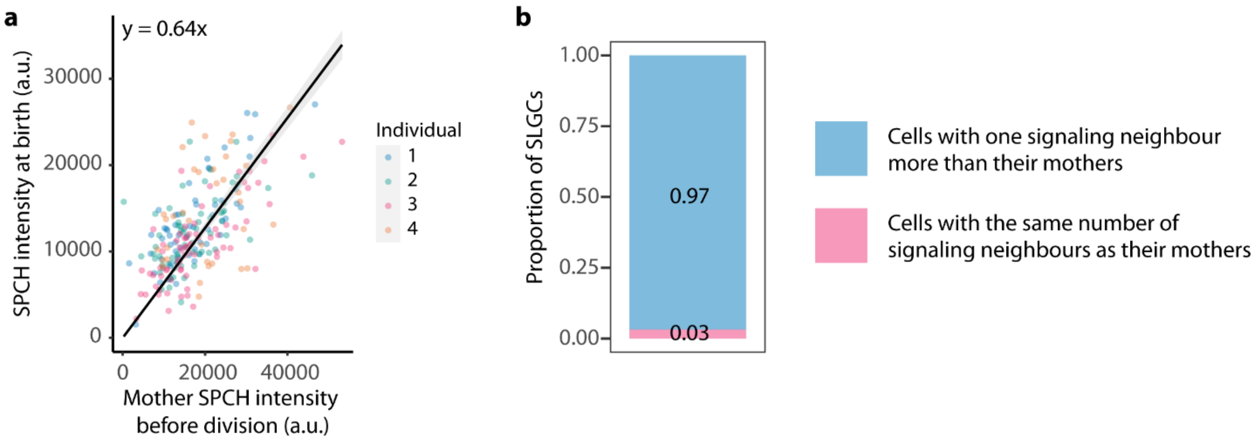

Supplementary Fig. 6 | SLGCs inherit approximately two-thirds of their mothers' SPCH intensities and have one signaling neighbour more than their mothers. a SPCH intensity at birth vs. mother SPCH intensity before division. The black line and grey band are linear model predictions and 95% confidence intervals. N = 4 individuals; 50, 62, 75, 75 cells per individual. **b** The proportion of SLGCs with one signaling neighbour more than their mothers (blue) and the same number of signaling neighbours as their mothers (pink). N = 2 individuals; 50, 75 cells per individual. Source data are provided in Source Data file 1.

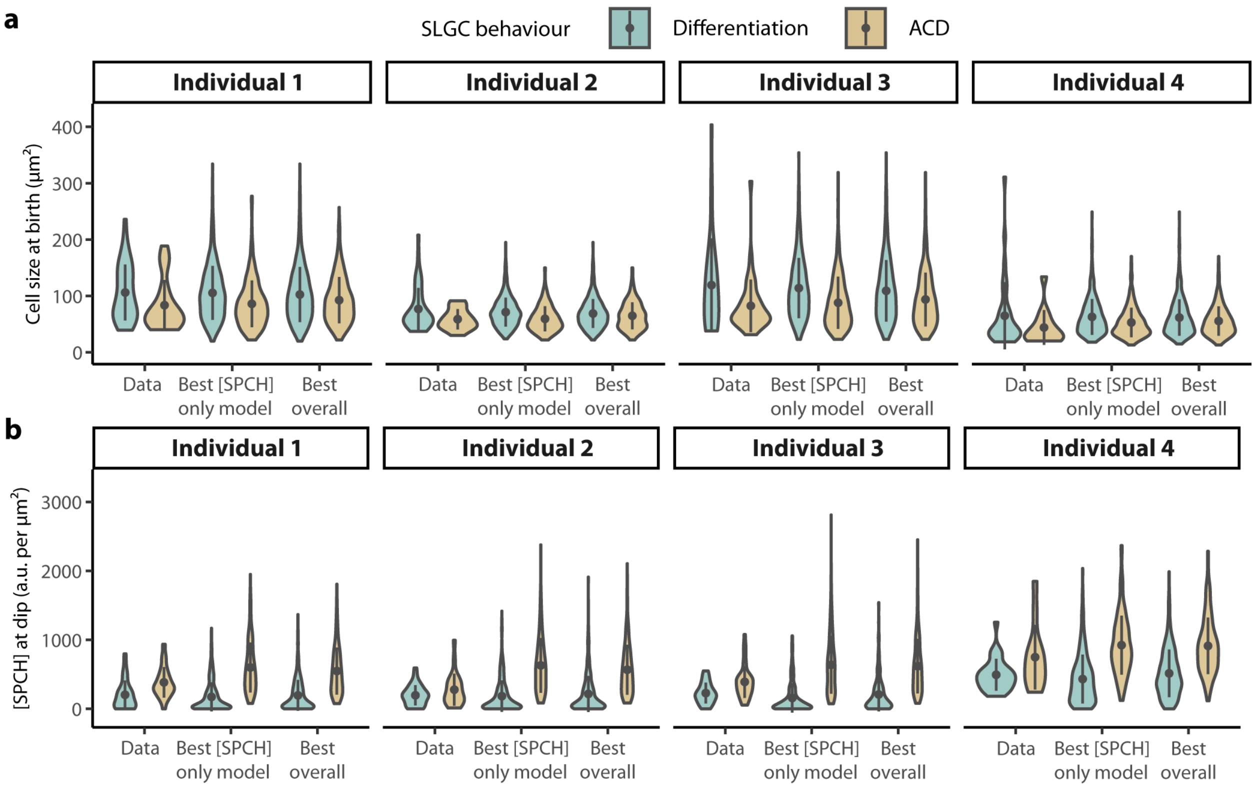

Supplementary Fig. 7 | The best [SPCH] only model and the best model overall can recapitulate the sizes and [SPCH] of dividing and differentiating cells. a-b The birth sizes (**a**) and [SPCH] at the dip (**b**) of dividing (ACD) and differentiating SLGCs in the data, compared to those in the best [SPCH] only model and the best model overall. In the best [SPCH] only model, degradation rates scale with neighbours and size, and [SPCH] at the dip is the sole determinant of SLGC behaviour. In the best model overall, degradation rates only scale with neighbours, and size, [SPCH], and neighbours are all considered in the fate-determining process. Data: $N = 4$ individual plants; 50, 62, 75, 75 cells per individual. Models: $N = 4$ individual plants; 1,000 cells per individual. Source data are provided in Source data file 1.

Supplementary Table 1 | Estimates of SPCH-YFP bleaching rates, compared to overall rates of decline in SPCH-YFP intensities from 0 to ~200 minutes after birth.

**Individual**	**Slope**	**Intercept**	**Bleaching rate (slope/intercept, per hr)**	**Mean decay constant (λ)**	**Overall rate of decline (% SPCH lost per hr)**
1	-235.61	14125	-1.67%	-0.649	-42.4%
2	-257.20	13163	-1.95%	-0.640	-44.0%
3	-339.80	15290	-2.22%	-0.404	-30.2%
4	-212.90	14647	-1.45%	-0.280	-22.7%

Supplementary Table 2 | Fitted parameters for simulations.
*Parameters for neighbour-based degradation. **Parameters for neighbour- and size-based degradation.

Variable	Distribution	Fitted parameters			
		**Individual 1**	**Individual 2**	**Individual 3**	**Individual 4**
Division asymmetry	Beta	$\alpha = 6.796$<br>$\beta = 14.278$	$\alpha = 9.706$<br>$\beta = 18.582$	$\alpha = 5.696$<br>$\beta = 13.385$	$\alpha = 5.850$<br>$\beta = 10.253$
Number of signaling neighbours of mother cell	Poisson	$\lambda = 0.597$	$\lambda = 0.507$	$\lambda = 0.467$	$\lambda = 0.200$
Mother cell size before division (μm^2)	Gamma	$\alpha = 5.033$<br>$\beta = 28.878$	$\alpha = 8.168$<br>$\beta = 12.561$	$\alpha = 3.668$<br>$\beta = 38.219$	$\alpha = 2.822$<br>$\beta = 28.414$
Mother SPCH intensity before division (a.u.)	Normal	$\mu = 17028.4$<br>$\sigma = 8213.8$	$\mu = 16819.4$<br>$\sigma = 7618.5$	$\mu = 17977.5$<br>$\sigma = 9631.3$	$\mu = 20549.3$<br>$\sigma = 8057.5$
SLGC growth rate (%/hr)	Mean	1.013	1.011	1.010	1.006
Randomly drawn degradation rates (λ)	Exponential	$\mu = 0.6644$	$\mu = 0.6410$	$\mu = 0.4193$	$\mu = 0.2823$
Degradation rates (λ) for cells with 1 neighbour*	Exponential	$\mu = 0.5417$	$\mu = 0.5878$	$\mu = 0.3429$	$\mu = 0.2494$
Degradation rates (λ) for cells with 2+ neighbours*	Exponential	$\mu = 0.7755$	$\mu = 0.7009$	$\mu = 0.5098$	$\mu = 0.4005$
Size-based, per-square-micron degradation rates	Exponential	$\mu = 0.0079$	$\mu = 0.102$	$\mu = 0.0056$	$\mu = 0.0068$
Size-based, per-square-micron degradation rates for cells with 1 neighbour**	Exponential	$\mu = 0.0075$	$\mu = 0.108$	$\mu = 0.0049$	$\mu = 0.0072$
Size-based, per-square-micron degradation rates for cells with 2+ neighbours**	Exponential	$\mu = 0.0082$	$\mu = 0.0096$	$\mu = 0.0063$	$\mu = 0.0052$
Duration of degradation (hr)	Mean	2.5574	2.7295	2.6667	2.2917

Supplementary Table 3 | Multiple logistic regression parameters for modes of fate determination.

Model	Terms	Estimate (*β*)
Size	Intercept	0.551870
	Size at dip	-0.011532
	Individual 2	-0.485926
	Individual 3	0.891775
	Individual 4	-0.819261
[SPCH]	Intercept	-1.5175689
	[SPCH] at dip	0.0033391
	Individual 2	-0.0210154
	Individual 3	0.7971070
	Individual 4	-1.3871696
Neighbours	Intercept	2.3762
	Signaling neighbours	-2.0042
	Individual 2	-0.3592
	Individual 3	0.8574
	Individual 4	-1.0107
Size + [SPCH]	Intercept	-0.4648068
	Size at dip	-0.0113864
	[SPCH] at dip	0.0034161
	Individual 2	-0.2722333
	Individual 3	0.8715108
	Individual 4	-1.8741598
[SPCH] + neighbours	Intercept	1.3555498
	[SPCH] at dip	0.0030930
	Signaling neighbours	-1.9100477
	Individual 2	-0.1847612
	Individual 3	0.8306865
	Individual 4	-2.0130550
Size + neighbours	Intercept	2.745461
	Size at dip	-0.005924
	Signaling neighbours	-1.855097
	Individual 2	-0.528401
	Individual 3	0.857530
	Individual 4	-1.233415
[SPCH] + neighbours + [SPCH]*neighbours	Intercept	1.4656494
	[SPCH] at dip	0.0027066
	Signaling neighbours	-1.9958963
	[SPCH] at dip*signaling neighbours	0.0002894
	Individual 2	-0.1856469
	Individual 3	0.8382843
	Individual 4	-1.9789828
Size + neighbours + size*neighbours	Intercept	1.876672
	Size at dip	0.003938
	Signaling neighbours	-1.209844
	Size at dip*signaling neighbours	-0.007204
	Individual 2	-0.496369
	Individual 3	0.878791
	Individual 4	-1.145140
Size + [SPCH] + neighbours	Intercept	1.7030774
	Size at dip	-0.0058269
	[SPCH] at dip	0.0030755
	Signaling neighbours	-1.7579984
	Individual 2	-0.3388473
	Individual 3	0.8447111

	Individual 4	-2.2166096
Size + [SPCH] + neighbours + size*neighbours + [SPCH]*neighbours	Intercept	1.0352947
	Size at dip	0.0034542
	[SPCH] at dip	0.0025846
	Signaling neighbours	-1.2775606
	Size at dip*signaling neighbours	-0.0066062
	[SPCH] at dip*signaling neighbours	0.0003713
	Individual 2	-0.3124349
	Individual 3	0.8679143
	Individual 4	-2.0895315
Size + neighbours + size*[SPCH]	Intercept	2.713
	Size at dip	-0.01677
	Signaling neighbours	-1.736
	Size at dip*[SPCH] at dip	0.00003119
	Individual 2	-0.3936
	Individual 3	0.8582
	Individual 4	-1.770

Supplementary Table 4 | Goodness-of-fit of each model to the proportion of SLGCs that divided given the number of signaling neighbours.

The top model, with the lowest corrected AIC (AICc) score, is highlighted in orange. Only models that passed equivalence and significance tests (see Methods) were evaluated.

**Model**		**Sum of squared errors (SSE), by individual**				**Corrected AIC (AICc)**
**Mode of fate determination**	**Mode of SPCH degradation**	**1**	**2**	**3**	**4**	
Size	Random	Failed equivalence and/or significance tests (see Methods)				N/A
	Neighbour					
	Size					
	Neighbour & size					
[SPCH]	Random	Failed equivalence and/or significance tests				N/A
	Neighbour					
	Size	0.153	0.176	0.465	0.106	-630.657
	Neighbour & size	0.150	0.182	0.444	0.122	-632.834
Neighbours	Random	Failed equivalence and/or significance tests				N/A
	Neighbour					
	Size					
	Neighbour & size					
Size + [SPCH]	Random	0.155	0.188	0.305	0.186	-647.662
	Neighbour	0.109	0.147	0.423	0.159	-648.262
	Size	0.133	0.186	0.488	0.158	-631.668
	Neighbour & size	0.129	0.197	0.457	0.189	-660.722
[SPCH] + neighbours	Random	Failed equivalence and/or significance tests				N/A
	Neighbour					
	Size	0.020	0.015	0.103	0.001	-852.691
	Neighbour & size	0.015	0.016	0.077	0.005	-874.761
Size + neighbours	Random	Failed equivalence and/or significance tests				N/A
	Neighbour					
	Size					
	Neighbour & size					
[SPCH] + neighbours + [SPCH]*neighbours	Random	Failed equivalence and/or significance tests				N/A
	Neighbour					
	Size	0.021	0.017	0.119	0.001	-835.531
	Neighbour & size	0.017	0.019	0.088	0.021	-845.500
Size + neighbours + size*neighbours	Random	Failed equivalence and/or significance tests				N/A
	Neighbour					N/A
	Size					N/A
	Neighbour & size					N/A
Size + [SPCH] + neighbours	Random	Failed equivalence and/or significance tests				N/A
	Neighbour	0.013	0.010	0.060	0.004	-903.265
	Size	0.024	0.017	0.121	0.000	-832.661
	Neighbour & size	0.020	0.019	0.090	0.005	-854.479
Size + [SPCH] + neighbours + size*neighbours + [SPCH]*neighbours	Random	Failed equivalence and/or significance tests				N/A
	Neighbour					
	Size					
	Neighbour & size					
Size + neighbours + size*[SPCH]	Random	Failed equivalence and/or significance tests				N/A
	Neighbour					
	Size					
	Neighbour & size	0.023	0.021	0.080	0.000	-860.574

Supplementary Table 5 | Newly generated and previously reported lines.

Genetic line	Sources
PlaCCI cell cycle reporter<br>*pATML1::mCherry-RCI2A*	This manuscript. We introduced a plasma membrane marker into the cell cycle marker line generated by Desvoyes et al. (2020).
pBRXL2::BRXL2-YFP<br>*pATML1::mCherry-RCI2A*	Gong et al. (2021a)
pATML1::H2B-mTFP<br>*pATML1::mCitrine-RCI2A*	Robinson et al. (2018)
spch-3<br>*pSPCH::SPCH-YFP*<br>*pATML1::mCherry-RCI2A*	Lopez-Anido et al. (2021)
spch-3<br>*pSPCH::gSPCH-YFP* (SPCH++)<br>*pATML1::mCherry-RCI2A*	This manuscript. We introduced a plasma membrane marker into a genomic SPCH reporter line that overproduces SPCH (Vatén et al., 2018).
pATML1::CYCD7;1-YFP<br>*pATML1::mCherry-RCI2A*	This manuscript. We transformed a wild-type line bearing a plasma membrane marker with the *pATML1::CYCD7;1-YFP* construct generated by Weimer et al. (2018).
spch-3<br>*pSPCH::SPCH2-4A-YFP*<br>*pATML1::mCherry-RCI2A*	This manuscript. We introduced a plasma membrane marker into the *pSPCH::SPCH2-4A-YFP; spch-3* line generated by Davies & Bergmann (2014).
