# Supplementary material for: Multi-scale dynamics influence the division potential of stomatal lineage ground cells in Arabidopsis
Source: Nat Commun. 2025 Mar 17;16:2612. doi: 10.1038/s41467-025-57730-9 (PMC11914061; doi:10.1038/s41467-025-57730-9)
Supplement: Supplementary file 2 — Reporting Summary [file 41467_2025_57730_MOESM2_ESM.pdf]

nature portfolio

Corresponding author(s): Dominique Bergmann
Last updated by author(s): 02/11/2025

Reporting Summary

Nature Portfolio wishes to improve the reproducibility of the work that we publish. This form provides structure for consistency and transparency in reporting. For further information on Nature Portfolio policies, see our Editorial Policies and the Editorial Policy Checklist.

Statistics

For all statistical analyses, confirm that the following items are present in the figure legend, table legend, main text, or Methods section.

n/a	Confirmed	
☐	☒	The exact sample size (*n*) for each experimental group/condition, given as a discrete number and unit of measurement
☐	☒	A statement on whether measurements were taken from distinct samples or whether the same sample was measured repeatedly
☐	☒	The statistical test(s) used AND whether they are one- or two-sided<br>*Only common tests should be described solely by name; describe more complex techniques in the Methods section.*
☐	☒	A description of all covariates tested
☐	☒	A description of any assumptions or corrections, such as tests of normality and adjustment for multiple comparisons
☐	☒	A full description of the statistical parameters including central tendency (e.g. means) or other basic estimates (e.g. regression coefficient) AND variation (e.g. standard deviation) or associated estimates of uncertainty (e.g. confidence intervals)
☐	☒	For null hypothesis testing, the test statistic (e.g. *F*, *t*, *r*) with confidence intervals, effect sizes, degrees of freedom and *P* value noted<br>*Give P values as exact values whenever suitable.*
☒	☐	For Bayesian analysis, information on the choice of priors and Markov chain Monte Carlo settings
☐	☒	For hierarchical and complex designs, identification of the appropriate level for tests and full reporting of outcomes
☒	☐	Estimates of effect sizes (e.g. Cohen's *d*, Pearson's *r*), indicating how they were calculated

Our web collection on statistics for biologists contains articles on many of the points above.

Software and code

Policy information about availability of computer code

Data collection	Image segmentation: ilastik (v1.4.0b15)
Data analysis	Plotting & statistical analysis: R v4.3.1<br>Simulations (lineage decision tree model): MATLAB 2021a<br>• code can be found on Figshare (doi: 10.6084/m9.figshare.27931686v.1)

For manuscripts utilizing custom algorithms or software that are central to the research but not yet described in published literature, software must be made available to editors and reviewers. We strongly encourage code deposition in a community repository (e.g. GitHub). See the Nature Portfolio guidelines for submitting code & software for further information.

Data

Policy information about availability of data

All manuscripts must include a data availability statement. This statement should provide the following information, where applicable:

- Accession codes, unique identifiers, or web links for publicly available datasets
- A description of any restrictions on data availability
- For clinical datasets or third party data, please ensure that the statement adheres to our policy

All data supporting the findings of this study are available within the paper and its Supplementary Information.

Research involving human participants, their data, or biological material

Policy information about studies with human participants or human data. See also policy information about sex, gender (identity/presentation), and sexual orientation and race, ethnicity and racism.

Reporting on sex and gender	N/A
Reporting on race, ethnicity, or other socially relevant groupings	N/A
Population characteristics	N/A
Recruitment	N/A
Ethics oversight	N/A

Note that full information on the approval of the study protocol must also be provided in the manuscript.

Field-specific reporting

Please select the one below that is the best fit for your research. If you are not sure, read the appropriate sections before making your selection.

[x] Life sciences   [ ] Behavioural & social sciences   [ ] Ecological, evolutionary & environmental sciences

For a reference copy of the document with all sections, see nature.com/documents/nr-reporting-summary-flat.pdf

Life sciences study design

All studies must disclose on these points even when the disclosure is negative.

Sample size	*Describe how sample size was determined, detailing any statistical methods used to predetermine sample size OR if no sample-size calculation was performed, describe how sample sizes were chosen and provide a rationale for why these sample sizes are sufficient.*
Data exclusions	Repeated imaging of seedlings must be done carefully to avoid damage and growth arrest. We only kept a time-lapse for analysis when meristemoids, SLGCs and GMCs underwent an ~equivalent number of cell divisions between 3-5 dpg as those classes of cells from sibling plants that had not been subjected to time-lapse imaging at 3-dpg, and the overall organ sizes of imaged and sibling plants were similar.
Replication	Experiments/analyses were typically replicated across 3-6 individuals to ensure reproducibility.
Randomization	Cells were sampled randomly, irrespective of behaviour.
Blinding	Investigators were blinded to the fate outcomes of each SLGC: specifically, features were measured at 3-dpg without knowledge of cell behaviours at 5-dpg. Blinding was not possible when comparing wild-type seedlings to pATML1::CYCD7, SPCH2-4A or SPCH++ seedlings, since these genotypes have very obvious phenotypes.

Reporting for specific materials, systems and methods

We require information from authors about some types of materials, experimental systems and methods used in many studies. Here, indicate whether each material, system or method listed is relevant to your study. If you are not sure if a list item applies to your research, read the appropriate section before selecting a response.

Materials & experimental systems

n/a	Involved in the study	
[x]	[ ]	Antibodies
[x]	[ ]	Eukaryotic cell lines
[x]	[ ]	Palaeontology and archaeology
[x]	[ ]	Animals and other organisms
[x]	[ ]	Clinical data
[x]	[ ]	Dual use research of concern
[ ]	[x]	Plants

Methods

n/a	Involved in the study	
[x]	[ ]	ChIP-seq
[x]	[ ]	Flow cytometry
[x]	[ ]	MRI-based neuroimaging

Plants

Seed stocks

Arabidopsis Biological Resource Center

Novel plant genotypes

Newly generated and previously reported lines are described in Supplementary Table 5. All transgenes used have been reported previously. Transgenic lines were generated by floral dip (Clough and Bent, 1998) and transgenic seedlings were selected on half-strength MS with the appropriate antibiotic (50 mg/L kanamycin or hygromycin). We analyzed 4-6 independent lines.

Generation experiments were performed

- PlaCCI cell cycle reporter; pATML1::mCherry-RCI2A (T3)
- spch-3; pSPCH::gSPCH-YFP (SPCH++); pATML1::mCherry-RCI2A (T3)
- pATML1::mCherry-RCI2A; pATML1::CYCD7-YFP (T2)
- spch-3; pSPCH::SPCH2-4A-YFP; pATML1::mCherry-RCI2A (T2)

Authentication

Transformed seedlings were screened for the appropriate fluorescent markers. They were also imaged to confirm the absence of a phenotype (for plasma membrane markers) or a hyperproliferation phenotype (for pATML1::CYCD7-YFP).
